# Advice for bad toxicologists^✰^

**DOI:** 10.1016/j.namjnl.2024.100002

**Published:** 2024-12-22

**Authors:** Richard R. Rabbit, aka Thomas Hartung

**Affiliations:** Johns Hopkins University, Center for Alternatives to Animal Testing (CAAT), USA, and University of Konstanz, CAAT-Europe, Germany

Congratulations! If you're reading this, you must aspire to master the subtle art of bad toxicology—a discipline steeped in tradition, often ignoring pesky modern science, and stubbornly clinging to the tried and outdated. With my foolproof advice, you'll never have to confront the challenges of real scientific progress. Let's dive in!

## 1 Stick to the ancient methods

Never question the tests developed in the 1970s and before. Why innovate when you can double down on high-dose animal testing protocols that predict human outcomes with barely the accuracy of a coin flip? Forget about mechanistic insights or human-relevant models; those are for the weak-hearted futurists. Remember: if it's not tested on a rat, it doesn't exist!

## 2 Worship at the altar of LD_50_

LD_50_—the test that determines the dose at which 50% of animals die—is your gospel. Who cares if it has little relevance to real-world exposures or human risk? Declare proudly: “*If 50% of rats died, it must mean something!*” Never bother with thresholds of toxicological concern or probabilistic risk assessments. The drama of death is far too exciting to relinquish.

## 3 Overlook reproducibility

Good toxicologists seek reproducible results, but not you! Reproducibility is for amateurs. Use minimal sample sizes and bypass statistical power analyses—these are mere distractions. Publish hurriedly; after all, who has time for careful validation when the next grant deadline looms?

## 4 Confuse complexity with precision

When presenting your findings, bombard your audience with graphs, p-values, and convoluted pathways that obscure your lack of actionable conclusions. Never simplify! The more impenetrable your presentation, the more impressed your audience will pretend to be.

## 5 Avoid human-relevant models

Why bother with *in vitro* systems, organoids, or microphysiological systems? Ignore their superior relevance to human biology and stick to your comfort zone. After all, developing alternatives requires effort, collaboration, and an open mind—things a true bad toxicologist would avoid at all costs.

## 6 Ignore the “3Rs”

The principles of Replacement, Reduction, and Refinement? Nonsense! Let those animal welfare activists argue all they want. A true bad toxicologist believes that more animals, higher doses, and unnecessary suffering somehow equate to better science. Never mind that public trust and funding are waning because of your archaic practices.

## 7 Refuse collaboration

Progress in toxicology thrives on interdisciplinary cooperation. Therefore, never consult computational biologists, engineers, or experts in systems biology. Ignore the potential of artificial intelligence in predictive toxicology—it's just a passing fad anyway!

## 8 Reject transparency

Keep your data opaque and your methods unpublished. Share only your conclusions, preferably in press releases touting breakthrough discoveries. This will save you from the embarrassment of having your results scrutinized, and you'll never have to face accusations of cherry-picking.

## 9 Stick to animal testing even when it fails

Even when animal models fail to predict human toxicity—as they do more often than not—insist on their validity. Argue that there's no alternative, even when there is. Dismiss systematic reviews that question the utility of animal models as mere academic nitpicking.

## 10 Treat every chemical equally

A bad toxicologist must never acknowledge differences in exposure, use cases, or populations. Whether it's a life-saving drug or a trivial cosmetic, apply the same outdated tests with the same irrelevant endpoints. Risk-based prioritization? Bah! All chemicals are equally guilty until proven innocent.

## Epilogue: a plea for reform

Of course, this advice is given with tongue firmly in cheek. The truth is, toxicology stands at a crossroads. Its practitioners have a profound responsibility—not only to protect human health and the environment but also to drive progress by embracing better tools and practices. Failure to adapt doesn't just harm science; it damages public trust, slows innovation, and perpetuates unnecessary suffering.

As Kurt Tucholsky might say, it's time for toxicology to wake up from its dogmatic slumber. Let us leave bad practices behind and strive for evidence-based, human-relevant, and ethical toxicology. The future demands it, and so should we.

## Declaration of competing interest

The authors declare the following financial interests/personal relationships which may be considered as potential competing interests: Editorial board member

## Data Availability

No data was used for the research described in the article.

